# Cardiology in a Digital Age: Opportunities and Challenges for e-Health: A Literature Review

**DOI:** 10.3390/jcm12134278

**Published:** 2023-06-26

**Authors:** Veronica Pegoraro, Chiara Bidoli, Francesca Dal Mas, Fabrizio Bert, Lorenzo Cobianchi, Maristella Zantedeschi, Stefano Campostrini, Federico Migliore, Giuseppe Boriani

**Affiliations:** 1 Governance and Social Innovation (GSI) Centre, Ca’ Foscari Foundation, 30123 Venice, Italy; veronica.pegoraro@unive.it (V.P.); chiara.bidoli@unive.it (C.B.); maris.zantedeschi@unive.it (M.Z.); stefano.campostrini@unive.it (S.C.); 2 Department of Economics, Ca’ Foscari University, 30123 Venice, Italy; 3 Department of Management, Ca’ Foscari University, 30123 Venice, Italy; francesca.dalmas@unive.it; 4 Department of Sciences of Public Health and Pediatrics, University of Turin, 10124 Turin, Italy; fabrizio.bert@unito.it; 5 Infection Prevention and Control Unit, ASL TO3 Hospitals, 10098 Turin, Italy; 6 Department of Clinical, Diagnostic and Pediatric Sciences, University of Pavia, 27100 Pavia, Italy; lorenzo.cobianchi@unipv.it; 7 Department of General Surgery, IRCCS Policlinico San Matteo Foundation, 27100 Pavia, Italy; 8 ITIR-Institute for Transformative Innovation Research, University of Pavia, 27100 Pavia, Italy; 9 Division of Cardiology, Department of Cardiac, Thoracic, Vascular Sciences and Public Health, University of Padua, 35122 Padua, Italy; federico.migliore@unipd.it; 10 Cardiology Division, Department of Biomedical, Metabolic and Neural Sciences, University of Modena and Reggio Emilia, Policlinico di Modena, 41124 Modena, Italy

**Keywords:** cardiology, telecardiology, telemedicine, heart diseases, literature review

## Abstract

To date, mortality rates associated with heart diseases are dangerously increasing, making them the leading cause of death globally. From this point of view, digital technologies can provide health systems with the necessary support to increase prevention and monitoring, and improve care delivery. The present study proposes a review of the literature to understand the state of the art and the outcomes of international experiences. A reference framework is defined to develop reflections to optimize the use of resources and technologies, favoring the development of new organizational models and intervention strategies. Findings highlight the potential significance of e-health and telemedicine in supporting novel solutions and organizational models for cardiac illnesses as a response to the requirements and restrictions of patients and health systems. While privacy concerns and technology-acceptance-related issues arise, new avenues for research and clinical practice emerge, with the need to study ad hoc managerial models according to the type of patient and disease.

## 1. Introduction

Cardiovascular diseases are the leading cause of death worldwide, accounting for 18 million victims annually. This number is forecast to rise and reach 24 million deaths annually by 2030, an estimated increase of 34% [1]. An inevitable consequence of this trend will be an overall increase in costs for health systems worldwide, which will surge from about USD 863 billion in 2010 to over USD 1 trillion. This trend is also reflected within the European continent, since cardiovascular diseases are responsible for 37.1% of all deaths, corresponding to approximately 1.7 million per year [2].

The primary cardiovascular diseases that can be traced back to this phenomenon include heart failure, stroke, and atrial fibrillation. In particular, heart failure is the leading cause of hospitalization in the over-65s. It is associated with very high mortality rates, as 1 in 25 patients does not survive the first hospitalization, and for survivors, a high re-hospitalization rate occurs in the first months post-discharge [3].

In this context, reducing mortality, increasing prevention, and improving the quality of life of people suffering from these diseases is a significant challenge for health systems worldwide. In addition, further critical issues make the situation more serious, such as the rising aging population, the shortage of health personnel, increasing healthcare costs, and the lack of congruence between investment needs and financing strategies [4,5]. In the last two decades and, more importantly, during and after the COVID-19 emergency, technology has increasingly been used as an instrument to face these challenges [5,6,7,8,9,10], thanks to its potential to expand the physical boundaries of healthcare systems, introducing the possibility to offer healthcare services remotely [4,11,12,13].

To date, many digital health technologies are available in the market, such as electronic decision-support tools, telemonitoring, remote monitoring, or mobile health applications (mHealth) [4,5]. Consequently, numerous studies have analyzed not only the advantages that these tools may bring to patients but also the disadvantages linked to their adoption and the barriers that need to be overcome to expand these technologies’ potential [4,5]; also considered is a virtual hospital model of care [5,14,15], in which a high number of patients can be telemonitored and assisted without being physically present in the ward. Digital literacy constitutes a potential barrier that has been the object of specific investigations, especially during the COVID-19 pandemic [16].

Coming to cardiovascular diseases, several e-health projects and trials are ongoing. For example, in the field of hypertension, the randomized, open-label HERB-Digital Hypertension 1 (HERB-DH1) trial carried out in Japan in 2021 demonstrated for the first time that digital therapeutics through a hypertension treatment app effectively lowered blood pressure in hypertensive patients [17]. The Japanese experience showed how patients in the digital therapeutics group who used the app and home blood pressure monitoring recorded lower blood pressure levels. The results of the pivotal study led to the first global approval of this app for the treatment of hypertension in Japan in 2022, along with reimbursement by medical insurance. Still, some issues emerge in identifying patients likely to respond to this therapeutic approach and developing clinical efficacy indices, calling for new guidelines for properly using hypertension applications in treating hypertension.

E-health is also being used to telemonitor and limit relevant risk factors for cardiovascular diseases such as diabetes. For instance, one American trial [18,19] aimed to assess the efficacy and safety of a digital therapeutic application delivering cognitive behavioral therapy designed to improve glycemic control in patients with type 2 diabetes. The positive results demonstrated how digital therapeutics might provide a scalable treatment option for patients.

Starting from these premises, the aim of our study is to present a systematic literature review [17] on the use of e-health solutions in the context of heart diseases to assess how the potential technology may contribute to solving or at least mitigating the grand challenges of the healthcare system. In addition, this paper aims to provide the reader with a framework to understand the current barriers to the adoption of health technologies and the aspects that need to be further investigated from a long-term perspective.

## 2. Materials and Methods

A systematic literature review was conducted [17] using PubMed and Scopus databases. The search string included the terms cardiology, e-health, telemedicine [(TITLE-ABS-KEY cardiology AND e-health OR telemedicine) AND (LIMIT-TO (DOCTYPE, ‘re’))]. From this process, 207 results were obtained. Afterward, all the studies published before 2018 were excluded, given that the rapid evolution of technological tools might have affected the analysis results. The studies considered were reduced to 109.

From reading the titles and abstracts, we excluded off-topic studies, such as those concerning pediatric patients and patients undergoing rehabilitation but not subject to a telemonitoring program, and studies focusing on the exclusive use of telemedicine solutions in the pandemic period.

**Figure 1:**
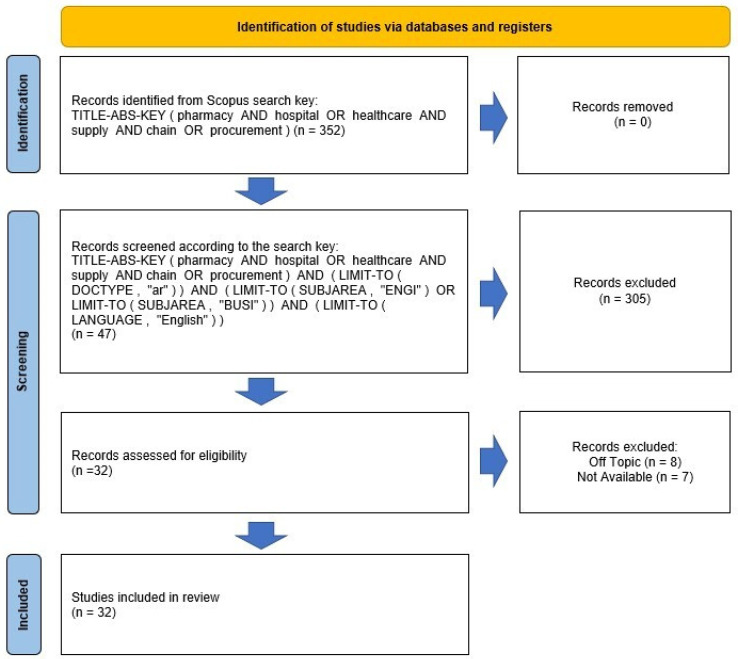
Flow chart of the revision steps according to the PRISMA protocol.

Furthermore, articles containing few details and data concerning key aspects of the present study, such as advantages and barriers in the application of e-health in the field of heart disease, were excluded. The selection resulted in 28 papers. Of the 28 studies, only 20 were finally coded, as it was not possible to retrieve the full text of 8 of them. Figure 1 reports the selection process according to the PRISMA framework [18,19,20].

The following Table 1 reports the 20 articles included in the literature review and their bibliographic data.

The selected documents were then read and coded by NVivo 12 software, considering as a framework of reference the nodes and subnodes usually reported in the recent literature [5,40,41,42] but adapting them to the purpose of the present study.

The first group of nodes focused on the context, such as the geographical area, the elements that facilitated the spread of e-health tools, and the main reasons for adopting them. The second category of nodes focused on the elements that helped to identify the topic framework, that is, the status of the patient (in follow-up or not), the type of clinical staff involved, and the general feedback collected, as well as the main tools and software used by the patients and medical staff. The third group referred to the potential of the technologies considered. In this regard, we analyzed the advantages, disadvantages, and barriers of this digital model of care. Finally, the fourth category focused on future perspectives, assessing which are the next challenges and objectives.

## 3. Results

### 3.1. Contextual Elements

Analyzing the geographical areas covered by the studies, it should be noted that most of the cases refer to the non-European context (no. 11), with specific reference to the USA and Canada, although there is also a notable match in the European Union framework (no. 5). Only four articles do not refer to any specific geographic context.

Coming to the specific content of the papers, some common elements can be discerned that encouraged the use of new technologies in the field of heart disease, as described in Table 2 below.

### 3.2. Ideal Patient Setting, Clinical Staff, and Technologies Used

#### 3.2.1. Ideal Patient Setting

In the considered studies, from the point of view of the considered pathologies, the area of patients adhering to telemonitoring and telecare programs is homogeneous. In particular, heart failure is one of the pathologies for which the use of telecardiology has been found to have more significant positive effects, such as the reduction of readmissions [27].

Furthermore, hypertension and arrhythmia are other widespread diseases in the analyzed population for which these programs are considered adequate; in fact, there is not an ‘ideal patient profile’. People who live in remote areas, anyway, are recognized to be key beneficiaries of this digital model of care [43].

Considering patients’ ages, it has to be highlighted that most of the studies do not make specific reference to an age group. However, in those in which age is presented, patients are usually over 60 [37]. However, marked differences may exist among patients over 60 with regard to digital literacy [16], which may act as a major determinant of the effective implementation of models of care based on digital tools.

#### 3.2.2. Clinical Staff

Results underline how the clinical team in charge of monitoring patients requires a multidisciplinary approach to guarantee a rapid and effective inclusion of patients in specific programs [36]. This team is, in most cases, composed of cardiologists, specialized nurses, pharmacists, and assistants who must necessarily collaborate and coordinate with the social and healthcare personnel at the territorial level, as well as the relevant administrative staff, to ensure the effectiveness of the model [36].

The presence of highly specialized staff continues to occupy an important position in the management of patients, despite the use of increasingly advanced technological equipment (such as artificial intelligence and automated software). This is due to the crucial role they play in improving the delivery of patient care. In fact, the activities of the medical personnel are not limited to the mere collection of vital parameters. On the contrary, they aim to positively influence the patient’s self-empowerment and psychological state [11].

From the perspective of the healthcare personnel involved in e-health programs, the overall feedback proves to be positive, given the reliability demonstrated by the devices in the collection and transmission of data. The pandemic contributed positively to this aspect by stimulating the spread of e-health, consequently increasing the awareness and credibility associated with such solutions [35]. However, the issue of appropriate reimbursement results as a key factor for the involvement of physicians in models of care based on the widespread use of digital tools, requiring time and dedication, coupled with professional responsibilities [45].

#### 3.2.3. Technologies Used

The digital tools that are used today by clinical teams for data collection and management are numerous. There are, in fact, no single applications or devices that have become mainstream. The analyzed studies focused mainly on telemonitoring. Considering this specific aim (which represents only one application in the broad e-health scenario), two major categories can be recognized: wearables and implantable devices [24]. The first group includes smartwatches [25], wearable biosensors [23], and pedometers [24]. The second group comprises defibrillators [44], pulmonary artery pressure monitoring devices, and pacemakers [24]. Device data may require combined use with other instruments, usually mobile, such as smartphones and portable ECGs for data collection and transmission [25]. In such a heterogeneous scenario, it is necessary to distinguish between applications and tools that obtained formal approval as medical devices, of which reliability in specific settings is proven, and other devices or apps of unproven value for medical purposes, frequently developed in the field of wellness [46]. The type of device also affects its use by patients, which can be under control and advice by physicians and medical personnel, but can also be, with increasing frequency, consumer-driven, without specific medical control [47].

The type of data transmitted and the frequency of transmission depend on the type of device, the indication for the specific implant (secondary or primary prevention), and the clinical status of the patient [25]. The patients’ parameters measured were very similar in all the studies examined, such as body weight, blood pressure, heart rate, blood glucose, oxygen saturation, and left atrial pressure [24]. The data collected by digital tools are then transmitted through specific apps and software. In this regard, most of the authors pointed out that it is crucial for the system’s actors to be equipped with an adequate and integrated information system capable of ensuring the effective and fast transmission of data [24].

The high speed of data transmission is essential for alerting cardiologists and other members of the clinical staff in case of need [24]. For example, when deviations from the standard vital values of patients are detected, an alarm is sent by the wearable device worn by these patients.

The use of smartwatches, and in general of small wearable devices, is generally more appreciated by patients than less discreet devices, which may interfere with the “normal conduct of daily life” [37]. In addition, although overall, patients prove to be satisfied with the use of new technologies [36], in some cases, they raised concerns because of the replacement of traditional care provided by clinical professionals with that offered by impersonal technologies [37].

### 3.3. The Benefits of Telecardiology

In the paper analysis, both advantages and disadvantages associated with the implementation of this new model of care have been highlighted. In particular, the following section reports the benefits that telecardiology brings to the lives of patients and health professionals, as well as within the community and the socio-medical system as a whole. Benefits have been divided into three macro-categories, as reported by the following Table 3.

#### 3.3.1. Clinical Management

Digital tools offer the opportunity to optimize care activities and services while increasing their quality. Indeed, they provide the possibility for analyzing massive amounts of data within a short time, consequently improving prevention, decision-making processes, and cardiac diagnostics [26].

The increased and constant use of telemonitoring tools may lead to a reduction in the number of unscheduled visits, as well as a decrease in admissions and readmissions to healthcare facilities, thus reducing the workload of cardiologists [25,44]. This allows for a better allocation of scarce human resources as well as an increase in standards of care and accessibility for a greater number of people, especially those living in remote areas [25].

Finally, the analysis of the papers showed that creating standardized and centralized governance for each observed pathology allows for more proactive management of patients [36]. The study conducted by Gabriel Sayer et al. [36], aimed at implementing a centralized management of heart failure patients, indicated as key steps for its design the identification of patients, the creation of a platform for organizing both patients and activities, the design of management protocols, and the recruitment of a centralized team. The identified patients receive a remote management kit including scales, blood pressure cuffs, and Bluetooth pulse oximeters at home.

#### 3.3.2. Technology, Data, and Costs

Technological progress today offers essential opportunities. Innovations such as artificial intelligence (AI), as well as advances in home automation in healthcare, make it possible to improve preventive medicine thanks to the use of algorithms that enable sophisticated data processing, as well as machine learning technology, which makes it possible to increase the reliability of the reports drafted [25,48]. However, already today, technology has proved to be sufficiently reliable for both diagnosis and patient monitoring [44].

From the cost point of view, there are several studies that have reported benefits for both the healthcare system as a whole and the patients. With regard to the first, the cost-benefit can be attributed, on the one hand, to a reduction in costs related to on-site care and infrastructure expenses [27] and, on the other hand, to cost savings due to increased prevention and the consequent decrease in readmissions and hospitalizations, as well as in recovery time [37]. Furthermore, it has been shown that a reduction in costs is associated with using electronic consultations, which are less time-consuming and more efficient. In fact, they take less time and allocate human resources better compared to standard consultations [25].

Several studies have also highlighted that the reduction in costs may be traced back to a decrease in patients’ travel expenses and time [23,44].

#### 3.3.3. Professionals and Patients

The greatest benefits indicated by the majority of the papers relate to the positive impacts that e-health brings to healthcare personnel and patients’ lives.

From the perspective of clinical organizations, it has been found that there is an improvement in the allocation of resources and in the communication between the members of care teams, and that there is an increase in transparency [36]. As a result, the workload is better distributed among caregivers [37], who can devote their attention to cases that require specific care and prompt intervention [27].

These factors not only alleviate the stress burden on both operators and healthcare systems but also improve decision-making processes, given that the clinical personnel may easily access patients’ medical records, which contain the complete patient medical history [34].

For what concerns the patients, all studies show an overall improvement in the quality of their life because of the increased level of comfort and reduced hospitalization readmission rates as well as mortality [31].

Patients claimed to feel more comfortable because they can continue to work without being interrupted by frequent in-person visits [23], but also because they can easily maintain interpersonal relationships with the medical staff, which telemedicine solutions are equally able to guarantee if compared to the traditional care model [11]. Enjoying a video connection has proved to be invaluable not only for monitoring vital parameters but also for offering psychological assistance, giving reassurance, and alleviating the sense of abandonment, isolation, and fear from which patients may suffer [36].

Televisits are also very valuable for healthcare professionals to check patients’ general level of physical or emotional distress, e.g., the onset of depressive symptoms, and their nutritional habits [11]. Moreover, the possibility of remaining in the home environment has proven to be more comfortable for many patients. Indeed, during teleconsultations, they can enjoy the presence and the help of their family members, who often cannot be present during hospital visits due to logistical and time issues [32].

With regard to the latter issue, time and cost savings represent fundamental aspects [23], especially for people living in rural areas, who can minimize the travel time to reach healthcare facilities for specialist care and follow-up consultations [23,33,44]. In addition, this digital model of care may also bring a reduction in socioeconomic disparities thanks to the user-friendliness of technological devices and their relatively low costs related to their widespread adoption [37].

The evolution of technology has enabled not only improvement in patients’ quality of life but also provoked a shift in patients’ role, from being passive subjects to being actively involved in the control of their health status and lifestyle [11]. In other words, the self-empowerment of the patients has increased because they can be directly involved in their treatment and monitoring and also in dialogue with healthcare professionals while enjoying greater decision-making power [11,30].

According to several studies, such involvement raises the rate of adherence to the therapy and positively impacts cardiac rehabilitation, reducing symptoms, improving the psychological status of patients, and reducing their mortality rate [26].

Finally, telecardiology plays a key role in educating patients, improving their compliance with medical treatments, and guiding them toward the adoption of healthy lifestyle habits [27].

### 3.4. Barriers and Limits of Telecardiology

The perceived barriers and limitations identified in the analyzed studies are mainly attributable to four macro-areas: governance, legislation, and policies; infrastructure, technology, and data; cost and investment; and health workforce and patients, as reported in the following Table 4.

#### 3.4.1. Governance, Legislation, and Policies

In most of the studies, it is pointed out that the lack of clear national standards [26] and guidelines [48] relating to the development of e-health poses important barriers to the spread of this new model of care. Furthermore, there is a lack of transparent regulation on the management of data collected from patients, ethical standards on their use [35], and common reference values for monitoring the effectiveness of given technologies [33]. These factors lead to an inevitable slowdown in the deployment of e-health, creating scepticism and mistrust among both patients and healthcare professionals [27].

With regard to guidelines, it has not been appropriately defined what clinical decisions are indicated in response to specific alarms related to preclinical changes in health status detected by implanted device diagnostics, such as may occur in heart failure [49]. Indeed, there is a need for more high-quality clinical studies to validate specific clinical pathways in response to the information provided by devices to be implemented in guidance documents for appropriate clinical decision-making in different settings. The lack of well-defined responses to specific signals derived from remote monitoring could explain the variable results obtained in particular settings such as heart failure [45,46].

Regarding legislation, the papers highlight that the norms to regulate and manage the digital model of care are inadequate, since they are limited to working with the traditional medicine model [25]. This can be a critical obstacle to the effective use of these devices by patients, who have highlighted, in some studies, their reluctance to provide their data due to the lack of guarantees and clear universally applicable standards [25].

#### 3.4.2. Infrastructure, Technology, and Data

The quality of the data is a key aspect to be taken into account in order to implement an efficient e-health model. For this reason, several authors have mentioned as a major limitation the lack of standardized and accurate methods for data collection [26] as well as poor patient engagement [25]. The latter, on the other hand, proves to be indispensable given the crucial role the patient plays in the collection and transmission of information [28]. In fact, the studies highlight the vast heterogeneity of the technologies, interventions carried out, and communication methods, as well as the different involvement of healthcare professionals and the follow-up parameters monitored, implying an insufficient standardization of telemedicine [29,30].

In addition, the reliability of the data depends not only on their consistency but also on the quality of the technologies used and the algorithms applied for the processing, which affect the quality of the final data collected [25].

Moreover, given the recent introduction of many of the devices used, false alarms are not uncommonly reported, affecting the workload of professionals who are overwhelmed by the amount of data to be analyzed [28]. An example reported by Sashini et al. [28] concerns a pilot telemonitoring program in which patients were divided into two groups, namely, heart failure and acute myocardial infarction, for the purpose of collecting and comparing alarms generated by the given devices. A total of 1094 alarms were reported, of which only 1% (10 alarms) were considered clinically significant.

A further obstacle to data transmission is the lack of integration of information flows. The lack of technical interoperability may, in fact, prevent the exchange of data between two or more technologies, such as between electronic health records and a new artificial intelligence application. Improving interoperability, on the other hand, is crucial to enable the training and dissemination of algorithms. Data scarcity is, therefore, a substantial obstacle to the scalability of technologies at a national level [24,29].

In addition, it is emphasized that the scarcity of national and institutional infrastructures dedicated to information and communication technologies (ICT) and digital tools are also key barriers to the implementation of the model. The absence or poor quality of the infrastructure needed to access the Internet, for instance, limits the access of inhabitants of many rural areas to data services [35,36]. In view of these limitations, there is a high risk of reaching the paradox that digital technologies and e-health are not accessible in the real world, to those categories of patients (older, living in rural areas, living in less developed and socially assisted contexts, etc.) for whom there is a gap of medical assistance in the traditional models of care delivery.

#### 3.4.3. Costs and Investments

In relation to the issue of costs, results are often uncertain. Some papers point out that the need to purchase specific technologies for obtaining and delivering care services leads to an increase in overall costs [44], while other authors argue that there is no real increase, but the costs incurred shift from the patient to the healthcare system [27]. Obviously, the time horizon, i.e., the period of time over which health outcomes and costs are calculated, stands as a crucial factor affecting the evaluation of investments and returns related to any initiative based on the implementation of digital technologies.

However, the lack of clear reimbursement pathways for the purchase of digital technologies and medical devices is considered a major barrier. In fact, as these instruments do not fall into the categories of traditional medicine, they are not formally considered medical devices, and therefore the expense is borne by patients [24]. Thus, one ethical issue emerges, addressing whether, by advancing technology, we are promoting and improving the care of only those who can afford these technologies. Digital affordability stands as a relevant barrier in those countries or continents where people cannot afford smartwatches or pressure-monitoring devices.

The main issue resulting from this is the limited accessibility of these services to the less affluent population, who therefore do not adhere to or else abandon the programs offered by healthcare facilities [39].

For those digital tools that received formal recognition as medical devices, new forms of procurement and reimbursement, such as pay-per-performance or risk-sharing agreements, could be considered and implemented [50].

#### 3.4.4. Staff and Patients

With regard to the users of these technologies, the studies considered pay attention, albeit to varying degrees, both to healthcare professionals, in a less in-depth manner, and to patients, subject to closer analysis. Considering the former, it is often pointed out that e-health solutions tend to negatively affect their workload and responsibilities. In fact, the caseload increases due to the numerous tasks that have to be performed for remote patient care [39], which are added to the activities that are already carried out in healthcare facilities.

A further barrier is low digital literacy, both among patients (found in most studies) [51] and clinical staff, who should possess the necessary technical and digital skills to process, interpret, and store medical data correctly [24].

Additionally, advanced age and a low level of education in patients are often identified as potential obstacles to the widespread use of such solutions [25], as well as a source of anxiety and alienation [38] feelings that are exacerbated by the inherent impersonality of digital services [34]. In specific settings, such as heart failure, atrial fibrillation, and stroke, characterized by advanced age [51,52,53], frequently associated with frailty and disability [43], regarding the lack of digital literacy [54], the involvement of caregivers and family members may be an option to be considered.

Of note, different systems of remote monitoring may req\uire variable degrees of active patient involvement in monitoring the underlying cardiac disease. As a matter of fact, remote monitoring of heart failure through cardiac implantable electronic devices provides automatic transmission of device-detected data, to be eventually, but not necessarily, integrated by patient data [55], while the use of external devices may require the involvement of the patient or of a caregiver [51].

All these challenges are compounded by additional barriers that relate to the time factor. Indeed, the success of programs depends on patient adherence to the indicated therapies, which inevitably tends to decrease in the long term, leading to increased mortality rates [28]. It is also influenced by more strictly psychological factors, which are mainly attributable to the interference of monitoring and treatment programs with patients’ routines [38].

## 4. Discussion

Results underline the potential role of e-health and telemedicine in supporting new solutions and organizational models for heart diseases as a response to patients’ and health systems’ needs and constraints. In such a perspective, the development of different management models for telemonitoring should be considered, depending on the pathology observed. Several solutions and experiences are identified. Veenis J.F. et al. [48], for example, suggest the development of a model for assessing and monitoring heart failure according to a dual strategy: reactive on the one hand and active on the other. The former is used to prevent heart failure (HF) decompensations and, in case of the need for imminent HF-related hospitalization, to alert treating clinicians, who have a limited time to intervene. Instead, an active strategy can be used to assess an ideal target for each patient by setting up a therapy that takes into account the feedback provided by the telemonitoring system. This would improve the patients’ clinical status, ensuring more stable parameters and enhancing their survival and quality of life. Ideally, the reactive strategy should be coordinated from a centralized body, potentially at a national level, to allow for timely intervention, while the active strategy, consisting of optimizing the therapy, should be managed by local healthcare teams, who are in close contact with patients.

The potential advantages are counterbalanced by the presence of several barriers to the practical translation of such new solutions. Given the central role that e-health will play in healthcare systems in the future, it will be crucial to conduct further research into the effectiveness, safety, and reliability of the technologies used and deployable, as well as into the security of the devices and the governance of the data collected [26].

This last topic is very relevant in e-health and mHealth opportunities assessment. Previous studies, indeed, showed how most popular mHealth apps have poor data privacy, sharing, and security standards, despite the well-structured and extensive European Union General Data Protection Regulation [56]. On the other hand, privacy concerns may limit the use of these technologies. Pool et al. reported that patients can be worried about their privacy in home telecare, telemonitoring, and surveillance, especially when we talk about video recording, behavioral data, location data, and future use of data [57]. However, these concerns can be reduced by informed consent and other privacy protection practices, and new technologies can be accepted if the communication regarding their use is effective.

In addition, the development of further studies is suggested for the implementation of strategies to ensure treatment adherence and improve long-term therapy management, with particular reference to data transmission. Furthermore, a more timely and accurate use of the collected data is required, both for sending alarms and for the assessment by healthcare personnel of the need for intervention [28].

A further aspect of interest is staff and patient education, to ensure usability and technology acceptance. In this regard, e-learning programs are recommended as low-cost solutions that can increase digital literacy rates, as well as confidence in the use of e-devices, thus increasing the effectiveness of treatments and adherence to therapy [39]. Some of the training programs could in fact be dedicated to junior figures among the medical staff, with the aim of providing them with the necessary skills and decision-making abilities to operate, thus alleviating the workload placed on senior figures [32]. The engagement of scientific societies should also be recommended to provide clinicians with guidelines and recommendations also in the use of e-health solutions [1].

To this end, further investments should be promoted, both in research and in the acquisition of tangible and intangible assets (e.g., software tools and devices) to optimize the remote care of patients with cardiovascular diseases [32]. In this perspective, particular attention should be paid to AI and big-data-enabled solutions, which may soon integrate existing tools. Indeed, they could be employed both in training programs [58], given their potential to simulate actual human interaction, and in data processing [39].

From a future perspective, it is also recommended that further study be carried out by considering larger samples for investigation, to ensure higher data reliability as well as a better understanding of the optimal strategies and technologies for treating different types of heart disease [48]. In this regard, non-invasive telemonitoring devices might prove more suitable for the treatment of less symptomatic patients than invasive ones [48]. For future research, it may be important to incorporate several physiological variables together to ensure a more accurate evaluation of remote monitoring systems and to achieve the best accuracy in the prediction. These exogenous factors included the physical activity of the patients, their diet and medication habits, and device variability [28].

## 5. Conclusions

In concluding our study, although the articles on the subject of e-health applied to heart disease are numerous in the literature, data describing the benefits associated with given solutions, such as reduction in costs, hospitalizations, and readmission rates, should be enhanced from a quantitative point of view. Furthermore, it would be important to deepen and analyze data about the actual reduction in mortality rates as well as the reduction in costs, which depend on the nature of the healthcare system as well as the observation perspective adopted. For this reason, in the near future, it will be necessary to conduct further research and monitor the production of experiences in this regard, both nationally and internationally. In this way, it will be possible to understand the real potential of these technologies, fostering the development of new organizational models and optimal strategies so as to ensure both an overall improvement of the healthcare system and the health of its citizens.

As with all studies, our piece of research has several limitations. First of all, although selected in a rigorous manner, our article sample is limited. Moreover, the technological content of our research and the fast development in the scenario may limit the validity of our results. Further analysis, including more keywords or updating results by adding other sources, including published conference proceedings, may offer new insights to study such an exciting and topical theme.

## Figures and Tables

**Table 1:** Papers included in the study and their bibliographic data.

N.	Authors	Title	Year	Journal	Ref.
1	Mishra K., Edwards B.	Cardiac Outpatient Care in a Digital Age: Remote Cardiology Clinic Visits in the Era of COVID-19	2022	Current Cardiology Reports	[21]
2	Ghilencea L.-N., Chiru M.-R., Stolcova M., Spiridon G., Manea L.-M., Stănescu A.-M.A., Bokhari A., Kilic I.D., Secco G.G., Foin N., Di Mario C.	Telemedicine: Benefits for Cardiovascular Patients in the COVID-19 Era	2022	Frontiers in Cardiovascular Medicine	[22]
3	Kędzierski K., Radziejewska J., Sławuta A., Wawrzyńska M., Arkowski J.	Telemedicine in Cardiology: Modern Technologies to Improve Cardiovascular Patients’ Outcomes—A Narrative Review	2022	Medicina (Lithuania)	[23]
4	Kulbayeva S., Tazhibayeva K., Seiduanova L., Smagulova I., Mussina A., Tanabay S. at al.	The Recent Advances of Mobile Healthcare in Cardiology Practice	2022	Acta Informatica Medica	[24]
5	Mohammadzadeh N., Rezayi S., Tanhapour M., Saeedi S.	Telecardiology interventions for patients with cardiovascular Disease: A systematic review on characteristics and effects	2022	International Journal of Medical Informatics	[25]
6	Senarath S., Fernie G., Roshan Fekr A.	Influential factors in remote monitoring of heart failure patients: A review of the literature and direction for future research	2021	Sensors (Switzerland)	[26]
7	Veenis J.F., Radhoe S.P., Hooijmans P., Brugts J.J.	Remote monitoring in chronic heart failure patients: Is non-invasive remote monitoring the way to go?	2021	Sensors (Switzerland)	[27]
8	Kinast B., Lutz M., Schreiweis B.	Telemonitoring of real-world health data in cardiology: A systematic review	2021	International Journal of Environmental Research and Public Health	[28]
9	Faragli A., Abawi D., Quinn C., Cvetkovic M., Schlabs T., Tahirovic E., Düngen H.-D., Pieske B., Kelle S., Edelmann F., Alogna A.	The role of non-invasive devices for the telemonitoring of heart failure patients	2021	Heart Failure Reviews	[11]
10	Piskulic D., McDermott S., Seal L., Vallaire S., Norris C.M.	Virtual visits in cardiovascular disease: a rapid review of the evidence	2021	European journal of cardiovascular nursing: journal of the Working Group on Cardiovascular Nursing of the European Society of Cardiology	[29]
11	Khanna S., Harzand A.	Preventive Cardiology in the Digital and COVID-19 Era: A Brave New World within the Veterans Health Administration	2021	Healthcare (Basel, Switzerland)	[30]
12	Adam S., Zahra S.A., Chor C.Y.T., Khare Y., Harky A.	COVID-19 pandemic and its impact on service provision: A cardiology prospect	2020	Acta Cardiologica	[31]
13	Vervoort D., Marvel F.A., Isakadze N., Kpodonu J., Martin S.S.	Digital Cardiology: Opportunities for Disease Prevention	2020	Current Cardiovascular Risk Reports	[32]
14	Lotman E.-M., Viigimaa M.	Digital Health in Cardiology: The Estonian Perspective	2020	Cardiology (Switzerland)	[33]
15	Miller J.C., Skoll D., Saxon L.A.	Home Monitoring of Cardiac Devices in the Era of COVID-19	2020	Current Cardiology Reports	[34]
16	Sayer G., Horn E.M., Farr M.A., Axsom K., Kleet A., Gjerde C., Latif F., Sobol I., Kelley N., Lancet E., Halik C., Takeda K., Naka Y.	Transition of a Large Tertiary Heart Failure Program in Response to the COVID-19 Pandemic: Changes That Will Endure	2020	Circulation: Heart Failure	[35]
17	Tully J., Dameff C., Longhurst, C.A.	Wave of Wearables: Clinical Management of Patients and the Future of Connected Medicine	2020	Clinics in Laboratory Medicine	[36]
18	Woo K., Dowding D.	Factors affecting the acceptance of telehealth services by heart failure patients: An integrative review	2018	Telemedicine and e-Health	[37]
19	Treskes R.W., Van der Velde E.T., Schoones J.W., Schalij M.J.	Implementation of smart technology to improve medication adherence in patients with cardiovascular disease: is it effective?	2018	Expert Review of Medical Devices	[38]
20	Molinari G., Molinari M., Di Biase M., Brunetti N.D.	Telecardiology and its settings of application: An update	2018	Journal of Telemedicine and Telecare	[39]

**Table 2:** Common elements facilitating the implementation of e-health solutions in cardiology wards.

Common Elements	Description
Spread of heart disease and high mortality rate	Globally, a high mortality rate is associated with the increased incidence of heart disease in the world population. For example, heart failure remains the primary cause of death of patients globally [28,42]. In addition to this, the rate of comorbidities and chronicity is rising because of the aging of the world population [11].
COVID-19 pandemic	The outbreak of the COVID-19 pandemic posed major limitations during the 2020–2022 time frame. This led to a reduction in services offered to cardiac patients and diminished preventive diagnostic activities. The situation incentivized the use of new digital solutions to ensure adequate patient care, as well as continuity of care and the promotion of preventive actions. All these activities proved crucial for cardiac patients, given the higher risk factor associated with cases of COVID-19-infection [35]. Patients’ safety was therefore guaranteed by digital tools, which allowed the delivery of care while maintaining the physical distance required by restrictions [36]
Dissemination of technology	Over the past decade, the rapid evolution of technology has boosted the collection of clinical data as well as the development of different fields, such as clinical informatics, which should be intended as a discipline dedicated to organizing, understanding, and using data to improve health care and patient outcomes [43]. In addition, technological advances have made the exchange of information more rapid and frequent, a key factor in clinical management [37].
Clinical management	Telemedicine helps the clinical management of patients with cardiovascular disease, not only at the hospital level but also at the ambulatory care level, even when there are no in-person visits [37].
Costs and personnel	The use of e-health tools can help to address the structural shortage of healthcare workers [11], as well as help to reduce costs by decreasing the number of hospitalizations [44]

**Table 3:** Benefits of Telecardiology.

	Description
**Clinic Management**	
Visits	Reduction in the number of unscheduled visits.
Diagnostic	Acceleration and optimization of cardiac diagnostics.
Centralized model	More proactive management of patients.
Prevention and decision-making	Improved preventive medicine and decision-making phase.
**Technologies, data, and costs**	
Accuracy and reliability	Diagnostic accuracy considered sufficiently high by clinical staff.
Advancement of technology	Introduction of new technologies such as artificial intelligence to increase the potential of health technologies and the accuracy of the data collected.
Costs	Reduction in the cost of care for both patients and healthcare facilities.
**Patients and Personnel**	
Workload	Increased efficiency of clinical staff.
Patient empowerment	Direct patient involvement in prevention, monitoring, and treatment processes.
Comfort and quality of life	Increased quality of life and overall patient comfort.
Logistics and waiting times	Decreased logistical time associated with physical transfer to social-health facilities and reduced waiting time.

**Table 4:** Barriers to Telecardiology.

	Description
**Governance, legislation, and policy**	
National guidelines	Lack of national guidelines and e-health strategies focused on appropriate clinical decision-making on the basis of patient-collected data.
Ethical and privacy standards	Lack of clear ethical standards and guidelines for both the use of these technologies and the protection of patient privacy.
Monitoring and evaluation standards	Lack of clear monitoring and evaluation standards on the effectiveness, scope, and impact of interventions based on specific technological tools.
Legislation	Backward national legislation for the management of new e-health models.
**Infrastructure, technology, and data**	
Accuracy and reliability	Lack of accuracy, or errors in collected data.
Digital infrastructure	Lack of adequate national or regional digital infrastructures.
Quality of technology	Low quality of technology.
Interoperability	Poor interoperability between information systems.
Data management	National and international differences in data collection, storage, and definitions standards.
**Costs and investments**	
Device costs and reimbursements	High cost of devices and lack of clear reimbursement pathways for digital technologies.
Accessibility	Low Internet accessibility.
**Health workforce and patients**	
Workload	Increased workload for healthcare personnel.
Digital literacy	Low digital literacy and skills of both patients and health workers.
Acceptability	Increased anxiety and low acceptance and perceived effectiveness of the use of devices.
Routine and adherence to therapy	Decreased adherence to therapy in the long term.
Language	Reduced availability of translation languages.

## Data Availability

Data available from the corresponding author upon request.
